# Subunit disassembly and inhibition of TNFα by a semi-synthetic bicyclic peptide

**DOI:** 10.1093/protein/gzu055

**Published:** 2015-01-20

**Authors:** Stefan Luzi, Yasushi Kondo, Elise Bernard, Lukas K. J. Stadler, Marina Vaysburd, Greg Winter, Philipp Holliger

**Affiliations:** MRC Laboratory of Molecular Biology, Francis Crick Avenue, Cambridge CB2 0QH, UK

**Keywords:** bicyclic peptide, phage display, protein engineering, TNF

## Abstract

Macrocyclic peptides are potentially a source of powerful drugs, but their *de novo* discovery remains challenging. Here we describe the discovery of a high-affinity (*K*_d_ = 10 nM) peptide macrocycle (M21) against human tumor necrosis factor-alpha (hTNFα), a key drug target in the treatment of inflammatory disorders, directly from diverse semi-synthetic phage peptide repertoires. The bicyclic peptide M21 (ACPPCLWQVLC) comprises two loops covalently anchored to a 2,4,6-trimethyl-mesitylene core and upon binding induces disassembly of the trimeric TNFα cytokine into dimers and monomers. A 2.9 Å crystal structure of the M21/hTNFα complex reveals the peptide bound to a hTNFα dimer at a normally buried epitope in the trimer interface overlapping the binding site of a previously discovered small molecule ligand (SPD304), which also induces TNF trimer dissociation and synergizes with M21 in the inhibition of TNFα cytotoxicity. The discovery of M21 underlines the potential of semi-synthetic bicyclic peptides as ligands for the discovery of cryptic epitopes, some of which are poorly accessible to antibodies.

## Introduction

Aberrant inflammatory processes are increasingly recognized as drivers in many pathological processes ranging from rheumatoid arthritis to cancer. The proinflammatory cytokine tumor necrosis factor-alpha (TNFα) is a critical mediator of normal inflammatory responses but its overproduction can cause acute and chronic tissue damage (Tracey *et al.*, 2008).

Novel therapies including anti-TNF antibodies like Humira^®^ have significantly improved disease outcomes in patients suffering from a range of important inflammatory conditions including rheumatoid arthritis, psoriasis and Crohn's disease (Palladino *et al.*, 2003). Despite their success in the clinic, anti-TNF antibodies suffer from drawbacks, including high manufacturing costs and poor tissue penetration spurring the development of alternative TNF antagonist agents. Examples include the small-molecule binder SPD304 (He *et al.*, 2005), the peptide antagonists WP9QY (Takasaki *et al.*, 1997), the human ubiquitin derived F10 (Hoffmann *et al.*, 2012) or the DNA aptamer VR11 (Orava *et al.*, 2012).

Here we have investigated the potential of macrocyclic peptides (Gilon *et al.*, 1991; McGeary and Fairlie, 1998; Adessi and Soto, 2002; Marsault and Peterson, 2011; Baeriswyl *et al.*, 2012) as another alternative class of TNF binders. To this end, we initiated further development of a previously described semi-synthetic macrocycle discovery platform (Heinis *et al.*, 2009), whereby bicyclic peptides are formed on phage by covalent attachment of three reactive cysteines to an organic core.

## Materials and methods

### Library construction

Four individual libraries with different loop sizes ((loop 1 + loop 2): 6 + 6, 3 + 6, 6 + 3 and 3 + 3) as well as two library pools were constructed as described below. The first library pool contained libraries with loop sizes (loop 1 + loop 2) of 6 + 5, 6 + 4, 6 + 2, 5 + 5, 5 + 4, 5 + 3, 5 + 2, 4 + 6, 4 + 4, 4 + 3 and 4 + 2, whereas the second library pool contained libraries with loop sizes (loop 1 + loop 2) 5 + 6, 2 + 6, 4 + 5, 3 + 5, 2 + 5, 3 + 4, 3 + 2, 2 + 4, 2 + 3 and 2 + 2. Each loop size was encoded with individual primers (see below). For example for a 2 + 6 library fused to pIII of the pH phagemid vector (Hoogenboom *et al.*, 1991), the following two primers were used: Primer 1 (loop 1): 5′-GGG TCG CAG GTC TCA **GCA**C NN MNN ACA CGC TGC CAT GGC CGG CTG and Primer 2 (loop 2): 5′-G GGT CGC A GG TCT CAG **TGC** NNK NNK NNK NNK NNK NNK **TGT** GGC GGT TCT GGC GCT GAA AC (underline refers to *Bsa*I restriction site, encoded **Cys** residues) giving rise to library sequence NNK NNG TGC NNK NNK NNK NNK NNK NNK, followed by C-terminal linker sequence preceding pIII: GGC GGT TCT GGC GCT (GGSGA).

The following iPCR mastermixes were prepared: 10 µl of 5× GC buffer, 4 µl of 2.5 mM dNTP mix, 2 µl of 10 µM library primer 1, 2 µl of 10 µM library primer 2, 1.5 µl of DMSO and 28 µl of MPW. The mixtures were put on ice and 2 µl of pH phagemid ssDNA (11.8 ng/μl) together with 0.5 µl of F-540 Phusion hot start polymerase (Thermo Scientific) were added before the PCR program was initiated: (i) 98°C for 30 s, (ii) 98°C for 10 s, (iii) 67°C for 20 s, (iv) 72°C for 6 min, (v) repeat steps (ii)–(iv) for 10 cycles and (vi) 70°C for 10 min. The PCR products were purified with a QIAquick PCR Purification Kit (Qiagen), digested with *Bsa*I (New England Biolabs), purified once more with a QIAquick PCR Purification Kit and then ligated with T4 DNA ligase (New England Biolabs). After phenol:chloroform extraction and ethanol precipitation, the circular library DNA was forwarded to rolling circle amplification with phi29 DNA polymerase (New England Biolabs) and random DNA hexamers. After a total incubation time of 24 h at 34°C (the volume was doubled twice during this time), the unpurified samples were digested with restriction enzyme Bam HI-HF (New England Biolabs) and purified with a QIAquick PCR Purification Kit. After ligation with T4 DNA ligase and subsequent purification with a QIAquick PCR Purification Kit, the circular library DNA was electroporated into ER2738 cells (Lucigen) and cells were grown on large square TYE/1% glucose/100 µg/ml ampicillin (TYAG) plates at 30°C overnight.

### Phagemid rescuing and selection

We streamlined the phage selection protocol to allow automated selection using biotinylated antigen captured on magnetic beads (Dynabeads M-280 Streptavidin) using a BioSprint15 bead handling robot (Qiagen) for all blocking, binding, washing and elution steps (Konthur, 2010). To avoid the isolation of binders to the streptavidin capture agent, we used neutravidin-coated magnetic beads in the second round of all selections. Neutravidin-coated magnetic beads were prepared from Tosylactivated Dynabeads M-280 according to manufacturer's instructions (Life Technologies). The coating efficiency was determined by comparing biocytin samples that have been incubated and not incubated with NeutrAvidin-coated Dynabeads following the instructions of the Pierce fluorescence biotin quantitation kit.

Phage selections also utilized the previously described KM13 helper phage (Kristensen and Winter, 1998) with a trypsin-cleavable pIII, which greatly increases the selection efficiency by rendering ‘bald’ phagemid particles (comprising only helper-phage derived pIII and not displaying peptide) non-infectious. Trypsinization potentially provided further benefits by preferentially cleaving linear and misfolded peptides that failed to crosslink to the organic core. Finally, we used a *sup*E amber-suppressor strain of *E. coli* as a phage host, encoding NNK derived *amber* stop codons as glutamine (Q).

In detail, pools of library colonies were grown in 2 × TY/2% glucose/100 µg/ml Ampicillin to a density (OD_600_) of 0.3 after which an excess of KM13 helper phages was added to the cell broth. After co-infection (30 min at 37°C non-shaking and 30 min at 37°C 220 rpm), the cells were centrifuged, resuspended in 2 × TY/100 µg/ml Ampicillin/50 µg/ml Kanamycin and incubated at 37°C, 220 rpm shaking for exactly 12 h. The next day, the cell broth was centrifuged and the supernatant was precipitated with 20% PEG/2.5 mM NaCl. Centrifugation at 13 000 rpm pelleted residual bacterial cells and the samples were reduced with 200 µM tris-(2-carboxyethyl)-phosphine hydrochloride (TCEP) for 1 h at room temperature (RT) followed by incubation with 100 µM organic core (either 1,3,5-tris(bromomethyl)benzene core (TBMB), 2,4,6-tris(bromomethyl)mesitylene (TBMB-methyl) or 1,3,5-tris(bromomethyl)-2,4,6-triethylbenzene (TBMB-ethyl) in 10% acetonitrile for 1 h (TBMB and TBMB-methyl cores) or 4 h (TBMB-ethyl core) in a 30°C water bath. After another PEG-precipitation step, the pellets were resuspended in 700 µl PBS/0.1% Tween-20. For each round of semi-automated robot selection with a BioSprint15 workstation, streptavidin- (rounds 1 and 3) or neutravidin (round 2)-coated magnetic beads were washed with PBS/0.1% Tween-20 and then coated with tumor necrosis factor-alpha (TNFα; Gibco) in PBS/0.1% Tween-20 for 1 h at RT. After blocking in PBS/0.1% Tween-20/2% Marvel milk powder for around 50 min, trypsinized batches of phagemid particles were applied to the beads and incubated for 1 h at 37°C in a rotating hybridization incubator. Subsequently, non-binding phagemids were washed away and magnetic beads were added to mid-log TG1 bacteria for infection and plating. After three rounds of selection and screening of monoclonal phagemids, two highly conserved sequences appeared for the selection with the TBMB-methyl core.

### Infectivity measurement

A 10 × dilution series in 2 × TY was prepared with 10 µl initial phage solution. TG1-TR grown to an OD_600_ of 0.4 were added to each dilution step and incubated at 37°C for 1 h, non-shaking. Afterwards, 10 µl of each dilution step was transferred to dried TYE plates containing the appropriate antibiotic for selection. Plates were incubated at 37°C overnight and individual colonies were counted the next morning, and the number of colony forming units (CFUs) per milliliter of initial phage or phagemid containing cell broth was calculated.

### Enzyme-linked immunosorbent assay

One hundred microliters of 42.9 nM biotinylated antigen in PBS/0.1% Tween-20 were added to a well of a StreptaWell High-Bind (Roche) strip and incubated at RT for 1 h, shaking. The well was washed five times with PBS/0.1% Tween-20 and residual liquid was soaked on kitchen tissue. The well was blocked with 100 µl of PBS/0.1% Tween-20/2% Marvel by incubating at RT for 1 h, shaking. After washing as before, a defined amount of phagemid particles or Bicycle-MBP fusion constructs in 100 µl PBS/0.1% Tween-20/2% Marvel was added and incubated at RT for 1 h, shaking. The well was then washed five times as before. For the phagemid particles, 0.6 µl anti-M13-HRP monoclonal antibody (1:5000, GE Healthcare) in 100 µl PBS with 1% Marvel was incubated at RT for 40 min, shaking. For the Bicycle-MBP fusion constructs 0.03 µl anti-MBP monoclonal antibody (New England Biolabs) in 100 µl PBS/1% Marvel was incubated at RT for 40min, shaking followed by washing and incubation with 0.03 µl anti-mouse IgG HRP antibody in 100 µl PBS/1% Marvel at RT for 40min, shaking. Finally, the well was washed 10× as before. One hundred microliters of 1 × TMB substrate solution (Thermo Scientific) was added and the well was incubated for a few minutes until a blue color was developed. Fifty microliters of 1 M sulfuric acid were added to stop the reaction and the absorbance was measured at 450 nm/650 nm with a SpectraMax 340PC384 Absorbance Microplate Reader (Molecular Devices).

### Affinity maturation

Affinity maturation libraries were prepared exactly as before except that vector PEP48-pH was used instead of pH. For the semi-automated robot selection, all libraries were blocked in RPMI 1640 GlutaMAX medium with 10% fetal bovine serum and 5% Marvel and then incubated with biotinylated TNFα for 30 min at 37°C (Pulse) and subsequently incubated with a 2 × /5 × /10× molar excess of non-biotinylated TNFα for 30 min at 37°C (Chase) and capture on magnetic beads for another 30 min at RT. The beads were then washed and incubated with mid-log TG1 bacteria for infection and plating as before.

### Bicycle-MBP fusions

Screening by phage ELISA can rise to a number of artifacts, including avidity effects (leading to an overestimation of affinity), the potential formation of non-cognate peptide structures by crosslinking *in trans* either with co-displayed peptides or with pIII intradomain cysteines and finally context-dependent stabilization or alteration of peptide conformation through proximity effects or direct interactions with the phage coat. In order to eliminate these potential phage-related artifacts and identify the highest affinity binders, we chose to reclone the phage ELISA positives for recombinant monovalent expression in a different monovalent context as *mal*E fusion proteins (MBP). The pMAL-pIII vector (Kather *et al.*, 2005) system was chosen because it was designed for cloning selected peptide sequences from the New England Biolabs Ph.D. Phage Display libraries as N-terminal fusions to MBP (maltose binding protein, encoded by the *malE* gene). MBP is a monomeric protein devoid of cysteines (in the proximity of the N-terminus). We reasoned that this allow better affinity (or at least *k*_off_) ranking in ELISA as well as avoiding the formation of non-cognate crosslinks.

Peptide insert fragments were created by PCR of phagemid DNA with primers pESEagI (5′-GAG TCA C|GG CCG CGC CAG AAC CGC CAC A) and pESNdeI (5′-GAG TCA CA|T ATG AAA TAC CTA TTG CCT ACG GCA) with Super Tag Polymerase. The amplified insert fragments and the pMAL-pIII vector were digested with restriction enzymes *Eag*I-HF and *Nde*I. The purified constructs were then ligated with T4 ligase and transformed into NEB Turbo Competent *E. coli*. Individual clones were picked and grown in 5 ml LB/5% glucose/100 µg/ml Ampicillin at 37°C, 250 rpm overnight. A 1:100 volume was transferred to LB/0.2% glucose/100 µg/ml Ampicillin and cells were grown until an OD_600_ of 0.5–0.8 was reached. IPTG (0.3 mM) was added and the broth was incubated at 25°C, 250 rpm shaking for 16 h. The cell broth was centrifuged and the pellet was resuspended in 30 mM Tris/20% sucrose (pH 8.0) after which 1 mM EDTA was added and incubated for 15 min. The samples were centrifuged and the pellet was resuspended in ice-cold 5 mM MgSO_4_. After incubation on ice for 40 min and centrifugation, 20 mM Tris, pH 7.4, was added and the samples were purified on an amylase resin column with elution with 10 mM maltose monohydrate. The purified samples were reduced with 1 mM TCEP for 1 h at RT followed by purification with a BSA blocked PD-10 desalting column (GE Healthcare). Addition of 60 µM organic core in 20% acetonitrile and incubation for 1 h (TBMB and TBMB-methyl cores) or 3 h (TBMB-ethyl core) in a 30°C water bath resulted in modified Bicycle-MBP fusion constructs, which were further purified with a BSA blocked PD-10 column and concentrated with a 10K MWCO Amicon centrifugal filter (EMD Millipore). The final purified and concentrated samples were in 1 ml PBS.

### L929 experiment

To a Costar 3595 96-well flat bottom plate, 100 µl of 3 × 10^4^ viable L929 cells (in RPMI 1640 GlutaMAX medium with 10% fetal bovine serum and 100 U/ml penicillin and 100 µg/ml streptomycin) were added and incubated at 37°C, 5% CO_2_ for exactly 24 h. To 250 pg/ml (final concentration) TNFα, different concentrations of Bicycles, SPD304 or solvents were added and preincubated for 2 or 24 h at 37°C, 5% CO_2_ before 100 µl of the mixtures was added to each well together 50 µl of 1 µg/ml (final concentration) Actinomycin D. After incubation at 37°C, 5% CO_2_ for exactly 24 h, 90 µl medium was removed from each well, which was subsequently topped up with 50 µl of a XTT/coupling reaction mixture (300 µl coupling reagent per 15 ml XTT (Cell Proliferation Kit II, Roche)). The plates were incubated for around 5 h at 37°C, 5% CO_2_, after which the red signals were measured with an absorbance microplate reader at 475 nm/660 nm.

### Mass spectrometry

The samples were buffer-exchanged into 10 mM ammonia acetate and the volume was adjusted to 100 µl. The mass spectrometry measurements were carried out on an LCT benchtop orthogonal acceleration time-of-flight (oa-TOF) mass spectrometer (Micromass). The source temperature was 20°C and a standard electrospray source was used. The capillary voltage was 2700 V and the sample was delivered from a syringe pump running at 3 µl/min. The vacuum was changed by a speedy valve to give a reading of ∼6e6 in the TOF region. The data were combined, processed and deconvoluted using the transform function within Masslynx v4.1 (Waters, UK). Results are given as deconvoluted spectra.

### Light scattering

Size-exclusion chromatography coupled with multi-angle light scattering (SEC-MALS) measurements were performed using a Wyatt Heleos II 18 angle light scattering instrument and Wyatt Optilab rEX online refractive index detector. Detector 12 in the Heleos instrument was replaced with Wyatt's QELS detector for dynamic light scattering measurement. Samples of TNFα and bicycle peptide (100 µl) were resolved on a Superdex S-200 10/300 analytical gel filtration column (GE Healthcare) running at 0.5 ml/min in 0.22 µm filtered PBS buffer before passing through the light scattering and refractive index detectors in a standard SEC-MALS format. Protein concentration was determined from the excess differential refractive index based on 0.186 RI increment for 1 g/ml protein solution. The concentration and the observed scattered intensity at each point in chromatogram were used to calculate the absolute molecular mass from the intercept of the Debye plot using Zimm's model as implemented in Wyatt's ASTRA software. Autocorrelation analysis of data from the dynamic light scattering detector was also performed using Wyatt's ASTRA software and the translational diffusion coefficients determined were used to calculate the hydrodynamic radius using the Stokes–Einstein equation and the measured solvent viscosity of 9.3e–3 P.

### Analytical ultracentrifugation

A mixture of 10 µM TNFα ± 9 µM unlabeled bicycle (M21h) with 1 µM Cy5-bicycle (cy5-M21Q-SSS) was centrifuged at 45 000 rpm at 20°C after preincubation for 42 h at RT. C(s) distributions was determined using SEDFIT with a frictional ratio of 1.19. The frictional ratio was calculated using SOMO hydrodynamic modeling of a structure of human TNFα (pdb 4TSV). The solvent density and viscosity (*ρ* = 1.00534 g/ml and *η* = 1.002 mPa s) were calculated using Sednterp (Dr Thomas Laue, University of New Hampshire).

### X-ray crystallography

Recombinant human TNFα was purchased from Gibco (Catalog Number: PHC3013). Bicycle was dissolved in H_2_O and added to TNFα powder. The complex was incubated at RT for at least 2 days and was ready to be applied to crystallization experiment. Crystals of the M21h–TNFα complex were grown from condition 15 of CS Cryo screen (CS Cryo screen condition 15: 25.5% PEG 8000, 0.085 M Na cacodylate, pH 6.5, 0.17 M ammonium sulfate, 15% glycerol) (Hampton Research), crystallized in space group *P*3221, and diffracted and were refined to 2.95 Å. The asymmetric unit of the crystal consists of two copies of TNFα–bicycle complex at a ratio of 2:1.

Initial 100-nl crystallization trials were carried out using the MRC robotic system (Stock *et al.*, 2005). Crystallization plates were incubated at 20°C. Hexagonal-shaped crystals were grown from condition 15 of CS Cryo screen (Hampton Research). The optimized crystals were grown from the drop mixed 1 µl of TNFα complex and 1 µl of well solution and incubated over 1 month. Crystals of the M21h–TNFα complex crystallized in space group *P*3221, and diffracted and were refined to 2.95 Å. The asymmetric unit of the crystal consists of two copies of TNFα–bicycle complex at a ratio of 2:1. Crystals were soaked in the buffer containing well solution supplemented with 15% of additional glycerol before flash-frozen by plunging into liquid nitrogen. Diffraction data were collected from a single crystal on beamline I04-1 at Diamond Light Source, UK at 100 K and wavelength 0.98 Å. Data were integrated in iMosflm (Battye *et al.*, 2011) and scaled with Aimless (Collaborative Computational Project, 1994). The phase was solved by Molecular Replacement method using Phaser (McCoy *et al.*, 2007) with chain A of TNFα trimer (Chan and Anderson, 2006) as a search model. Four copies of TNFα monomer were found in an asymmetric unit. The bicycle model was manually built into the MR map using Coot (Emsley and Cowtan, 2004) and the structure was refined by Refmac (Murshudov *et al.*, 2011).

## Results and discussion

### Optimizing bicyclic peptide display

**Fig. 1.**
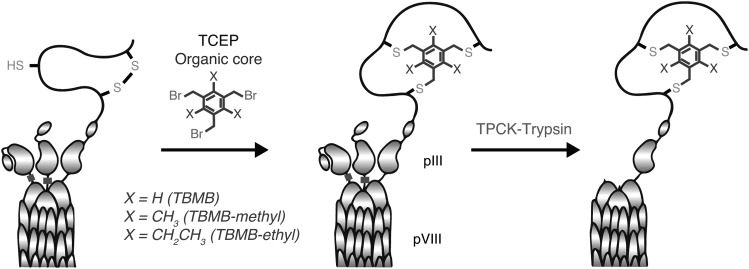
Phagemid display scheme and modification protocol. Phagemid display scheme: Phagemid particles are reacted with TCEP and organic cores (TBMB core, X = H/TBMB-methyl core, X = CH_3_/TBMB-ethyl core, X = CH_2_CH_3_) inducing on phage peptide crosslinking. Helper-phage pIII can be selectively cleaved with Trypsin and an intradomain K residue (black bar) to remove ‘empty’ pIII domains (Kristensen and Winter, 1998).

We first sought to improve both scope and efficiency of the original bicyclic peptide display strategy (Heinis *et al.*, 2009) by optimizing crosslinking chemistry and expanding chemical diversity from the original 1,3,5-Tris(bromomethyl)benzene core (TBMB) to two novel organic cores: 2,4,6-Tris(bromomethyl)mesitylene (TBMB-methyl) and 1,3,5-Tris(bromomethyl)-2,4,6-triethylbenzene (TBMB-ethyl). Using a bicyclic peptide binder (PEP48: CVRFGWTCDNSWHGC) of the MDM2 E3 ubiquitin ligase isolated by the standard method (Heinis *et al.*, 2009) as a benchmark, we optimized reaction temperatures, concentrations of the reducing agent TCEP and amounts (% v/v) of acetonitrile solvent. This yielded a superior protocol with much milder reaction conditions that no longer reduce intraprotein disulfide bonds. This allowed efficient on-phage peptide crosslinking chemistry even on phage particles with a wild-type pIII protein, enhancing post-modification titers of infective phage by >50-fold (Supplementary Fig. S1a and b). Furthermore, again using PEP48 as our model, we developed a phagemid system for the monovalent display of bicyclic peptides joined to the N-terminus of pIII via a flexible GGSGA linker and comprising a Trypsin cleavable pIII-variant helper phage eliminating background infectivity of ‘bald’ phages not displaying peptide (Kristensen and Winter, 1998) and reducing avidity effects of multivalent peptide display, which can hinder the isolation of high affinity binders (Fig. 1).

### Bicyclic peptide library construction and validation

Both antibodies and macrocyclic peptides show diversity in loop lengths. We sought to mimic this diversity in library design, devising a scheme for the rapid construction of bicyclic peptide libraries with variable loop sizes. Key steps involve inverse PCR (iPCR) amplification of phagemid with primer encoding library diversity, circularization of linear PCR products by ligation and rolling circle amplification (RCA) of ligated phagemids (Supplementary Fig. S2, Materials & methods) readily yielding libraries of 10^9^–10^10^ clones (CFU) (Supplementary Table S1). We randomized loop sequences and encoded all loop length combinations between 2 and 6 for a total phage repertoire size of 3.5 × 10^10^ CFUs. Functional diversity of the peptide library was further enhanced by peptide crosslinking to three different organic cores (TBMB, TBMB-methyl, TBMB-ethyl) yielding a potential combined chemical and biological diversity of 10^11^ different macrocycles.

We validated our system by selecting for bicyclic peptide binders to MDMX, a target protein closely related to MDM2 known to have yielded binders such PEP48 (see above) from the original 6 + 6 loop library and standard TBMB organic core (Heinis *et al.*, 2009). Our phagemid system yielded strong enrichment for all three organic cores over three rounds of selection as judged by the increase in binding activity of the polyclonal phage pool. Additionally, phagemid ELISA of individual clones showed specific binding to either MDMX or cross-reactive binding to both MDMX and MDM2 but no binding to unrelated proteins (Supplementary Fig. S3).

### Selection and affinity maturation of bicyclic peptide ligands against human TNFα

Encouraged by the ease with which we had obtained multiple ligands to MDMX and MDM2, we applied the system to Human TNFα (hTNFα), which had previously proved a challenging target for peptidic ligand discovery. Indeed, after just three rounds of selection, we observed a clear and specific rise in binding activity in the polyclonal phagemid pool for all three organic cores. Screening of individual clones by phage ELISA identified multiple strong hTNFα binders. In order to eliminate potential multivalent display related artifacts, i.e. avidity effects of rare multivalent phagemid particles, we rescreened phage ELISA positives as monovalent *mal*E fusion proteins (MBP) to identify the highest affinity binders (Supplementary Fig. S4a).

Rescreening of TNFα binders as MBP-fusions identified bicyclic peptides M8 (CPPCVWQALC)-MBP and M9 (CPPCVWQVFC)-MBP (both modified with TBMB-methyl cores) as the bona fide highest-affinity binders. M9-MBP proved highly specific with essentially no binding observed to different members of the TNFα superfamily (including the closely related TNFβ) or the unrelated protein MDM2 (Supplementary Fig. S4b) and chemically synthesized M9 bicyclic peptide effectively competed binding of the cognate M8/9-MBP-fusions (Supplementary Fig. S4c). Binding activity was also entirely dependent on the presence of the cognate TBMB-methyl core, with no TNF binding observed with either alternative cores or in the absence of organic core cross-linking (Supplementary Fig. S5).

Next we initiated affinity maturation of the consensus sequence ACPPC(L**/V**)WQ(**A/**V)(L**/F**)CGGSGA using two diversification strategies, either varying all amino acids (except C, the N-terminal A and C-terminal GGSGA linker) but restricting variation to mutations maintaining the physiochemical characteristics, or allowing all possible mutations at all positions (at 1.7 mutations per peptide). After four rounds of increasingly stringent selection, we isolated a range of closely related peptide sequences clustering around a consensus represented by peptide M21 (CPPCLWQVLC). Neither the N-terminal A nor the C-terminal GGSGA linker were mutated indicating that they are peripheral to binding activity.

### Characterisation of selected bicyclic peptides

We therefore prepared peptides by chemical synthesis and determined affinities using both fluorescence polarisation (FP) and microscale thermophoresis (MST). To disentangle the potential contributions of fluorophores and linkers to binding affinity, we prepared a range of different versions of the initial M9 and affinity-matured M21 bicyclic peptides comprising either Cy5 or 5,6-carboxyfluorescein (FAM) dyes at the N- or C-terminus as well as different C-terminal extensions (G, GGSGA and GGSGSGSG (to increase solubility)) (Table I) and measured their affinities by FP.

**Table I.** Fluorescence polarisation experiments. Affinity measurements by microscale thermophoresis (MST) confirmed FP results with Kd_MST_ values of 7–9 nM (Cy5-Ahx-Gly-M9) in excellent agreement to Kd_FP_ values for the same peptide (7.6 ± 1.0 nM). We continued further work with variants of the M21h peptide that displayed favourable physicochemical properties as well as the highest affinity.

Name	Sequence	Kd (nM)
M9C (Cy5-Ahx-Gly-M9)	Cy5-Ahx-GACPPCVWQVFCGGSGA	7.6 ± 1.0
M9dC (M9-diamino-Cy5)	ACPPCVWQVFCGGSGA-diamino-Cy5	12.5 ± 2.8
M21nl (Cy5-Ahx-Gly-M21-G)	Cy5-Ahx-GACPPCLWQVLCG	28.0 ± 3.8
M21 (Cy5-Ahx-Gly-M21)	Cy5-Ahx-GACPPCLWQVLCGGSGA	13.5 ± 2.7
M21h (Cy5-Ahx-Gly-M21G_2_(SG)_3_	Cy5-Ahx-GACPPCLWQVLCGGSGSGSG	5.3 ± 0.5
M25F (M25-Sar_6_-K(FAM))	ACPWCIWQPFCGGSGA- Sar_6_-K-FAM	6.7 ± 0.9

While we observed little difference in the binding affinity depending on either dye chemistry or position of attachment (N- vs. C-terminus), longer and more hydrophilic C-terminal linkers appeared to improve the binding affinity of the M21 peptide from 28 nM (for C-terminal G) to 5 nM (for C-terminal GGSGSGSG) (Table I). We also determined the affinity of another affinity-matured variant M25 (CPWCIWQPFC, with a 5,6 carboxyfluorescein attached to a lysine at the C-terminus = M25-Sar_6_-K(FAM) yielding a comparable *K*_d_ to M21 variants.

**Fig. 2.**
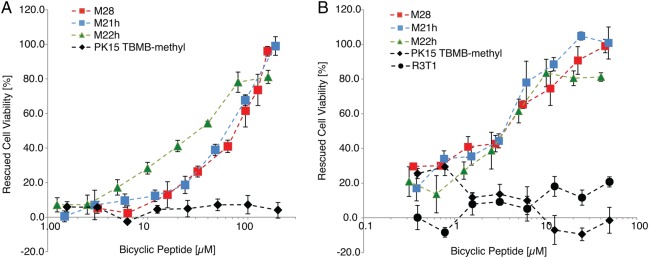
Cell survival experiments with the L929 bioassay. (A) Titration of bicyclic peptides to 250 pg/ml TNFα with 2 h preincubation at 37°C, 5% CO_2_. (B) Titration of bicyclic peptides to 250 pg/ml TNFα with 24 h preincubation at 37°C, 5% CO_2_. Bicyclic peptide concentrations are indicated on the *x*-axis. Rescued cell viability is indicated on the *y*-axis. Cell death was measured by monitoring residual cell metabolism using a colorimetric assay (tetrazolium salt XTT). Peptides are M21h: ACPPCLWQVLCGGSGSGSG and closely related variants M22: ACPWCPWQVLC, and M28: ACPPCPWQALC as well as control peptides R3T1: ACIRSLSCYILGCG, PK15: CSDRFRNCPADEALC (Heinis *et al.*, 2009).

Having established high-affinity binding to hTNFα, we tested whether the bicyclic peptides could inhibit TNFα effector functions using the well-established L929 fibrosarcoma necroptosis assay (Vanlangenakker *et al.*, 2011). Peptides M21h (M21 with a C-terminal GGSGSGSG linker) and M21hDpr (M21 with a C-terminal GGSGSGSG-diaminopropionic acid linker) yielded IC_50_ values of 66 and 115 µM respectively, with 99% cell viability rescued by 189 µM M21h (Fig. 2A). Longer pre-incubation of peptides with TNFα (24 h instead of 2 h) yielded a more than 10-fold lower IC_50_ value for M21h (IC_50_ = 4 µM) with full rescue of cell viability at 24 µM (Fig. 2B), suggesting an unusual, time-dependent mechanism of inhibition. We hypothesized that M21h (and M21hDpr) binding might induce changes in TNFα tertiary or quarternary structure reducing its bioactivity rather than directly antagonizing TNF R1 receptor interaction and signalling.

**Fig. 3.**
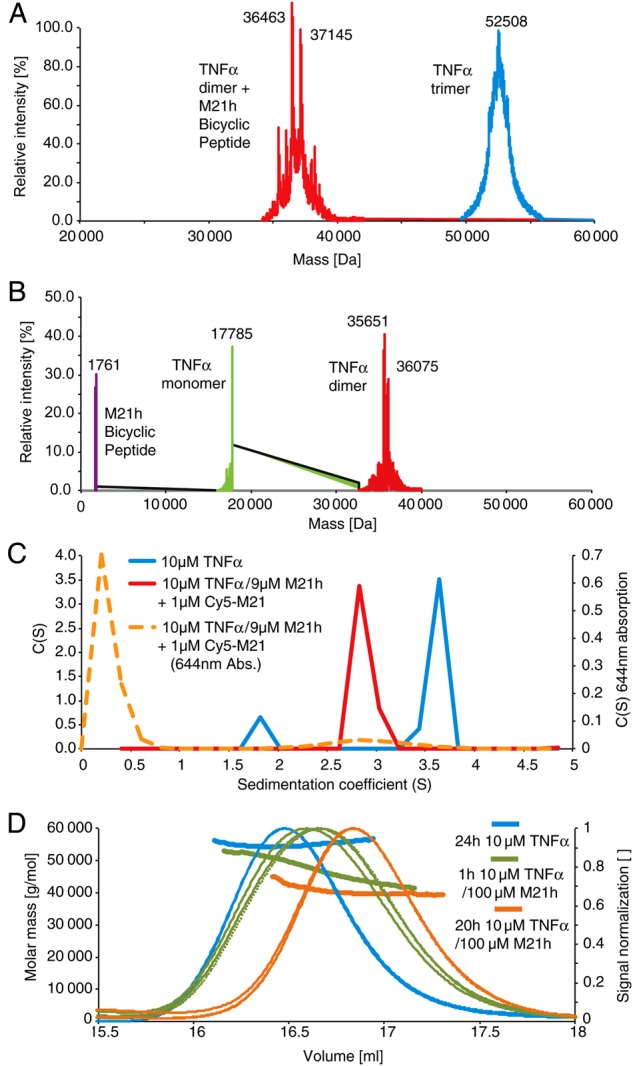
Mass spectrometry (MS), analytical ultracentrifugation (AUC) and light-scattering experiments (MALS). (A) LCT ESI-TOF MS: TNF-α (10 μM) without (blue) or with excess M21h (100 µM) incubated for (A) 4 weeks (RT) or (B) 1 h (4°C, PBS) (C) AUC: 10 µM TNFα with (red line) or without (blue line) unlabeled bicyclic peptide (M21h) and cy5-labeled bicycle (cy5-M21h) incubated for 42 h at RT before centrifugation at 45 krpm and sedimentation and sedimentation (D) SEC-MALS: TNFα (10 µM) with or without M21h pre-incubated for different times at RT.

In order to investigate the nature of the time-dependent effect on TNFα inhibition by M21, we analyzed M21h peptide-TNFα complexes using liquid chromatography electrospray ionization time-of-flight mass spectrometry (LCT ESI-TOF MS), analytical ultracentrifugation (AUC) and size-exclusion chromatography multi-angle static light-scattering (SEC-MALS). MS spectra of TNFα showed a clear peak at around 52,508 Da corresponding to the trimeric form and stable over a long timeframe (4 weeks at 4°C). However, after incubation with bicyclic peptide M21h two peaks of 36,463 and 37,145 Da form, corresponding to a TNFα dimer with and without bound bicyclic peptide (Fig. 3A). After longer pre-incubation, TNFα trimers are converted quantitatively into dimers and monomers by M21h (Fig. 3B).

AUC interference data (Fig. 3C) shows TNFα sedimenting as two species: a minor 1.9S and major 3.6S species, likely representing the TNFα monomer and trimer. Addition of the bicyclic peptide (red line) results in a shift of the majority of TNFα molecules to an intermediate species: 2.9S with the molecular weight expected for a TNFα dimer. Monitoring the sedimentation shows that the M21h peptide sediments quantitatively with the TNFα dimer with no free peptide observed, entirely consistent with the MS data.

SEC-MALS indicates that binding of M21h peptide results in the appearance of an apparent lower mass species (after 1 h) and shifting of elution volume after 20 h (40.3 kDa ± 1), both clearly distinguishable from native TNFα (54.5 kDa ± 0.5) (blue line) (Fig. 3D). Thus, all three methods independently and consistently indicate a time-dependent disassembly of TNFα trimers into dimers and monomers upon binding of the bicyclic M21 peptide.

A similar effect on TNFα structure had previously been described for the small molecule SP304. We therefore sought to investigate whether the combined effects of M21 bicyclic peptide and SP304 on TNFα structure and function would be competitive (suggesting identical or overlapping binding sites) or additive/synergistic (suggesting different (or only marginally overlapping) binding epitopes). SPD304 (He *et al.*, 2005) displayed an IC_50_ value of ca. 8 µM independent of preincubation but is unable to fully rescue cell viability (to >73%) due to significant toxicity at higher concentrations (100% cell death at >100 µM SPD304). This toxic effect of SPD304 may be explained by its 3-alkylindole moiety, which is bioactivated into a reactive electrophilic intermediate by cytochrome P450s and able to react with DNA and protein targets (Sun and Yost, 2008; Chan *et al.*, 2010).

**Fig. 4.**
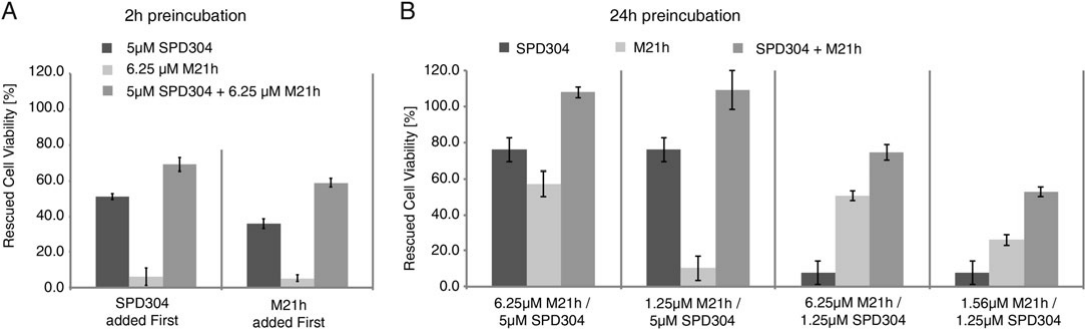
Cell survival experiments with the L929 bioassay. Different combinations of the small molecule SPD304 (He *et al.*, 2005) and M21h bicyclic peptide were applied to L929 mouse fibroblast cells with 2 h (A) or 24 h (B) preincubation. Rescued cell viabilities are indicated on the *y*-axis. Combined effects of M21h peptide and SPD304 small molecule between 12% and 19% higher than expected for simple additive effects in this concentration range indicating synergistic action on TNFα structure and function.

However, M21 bicyclic peptide and SPD304 combined can rescue 100% viability. Synergy between the two compounds was also evident for a 2 h preincubation experiment independent of order of addition. Indeed, all combinations of SPD304 and M21h yielded significantly higher cell viability in the L929 assay than with individual compounds alone (Fig. 4). These data suggest that SPD304 and the bicyclic M21 peptide bind to largely non- or weakly overlapping epitopes and together promote TNFα trimer disassembly.

### Structure of a bicyclic peptide-TNF complex

In order to better understand the effects of the M21 bicyclic peptide on TNFα structure and function we determined a structure of the peptide-cytokine complex by X-ray crystallography at 2.95 Å resolution. Crystals of the M21h-TNFα complex crystallised in space group P3221, and were refined to 2.95 Å (Supplementary Table SII and Fig. S6). The M21-TNF complex structure has been deposited in the Protein Data Bank as PDB entry 4TWT.

**Fig. 5.**
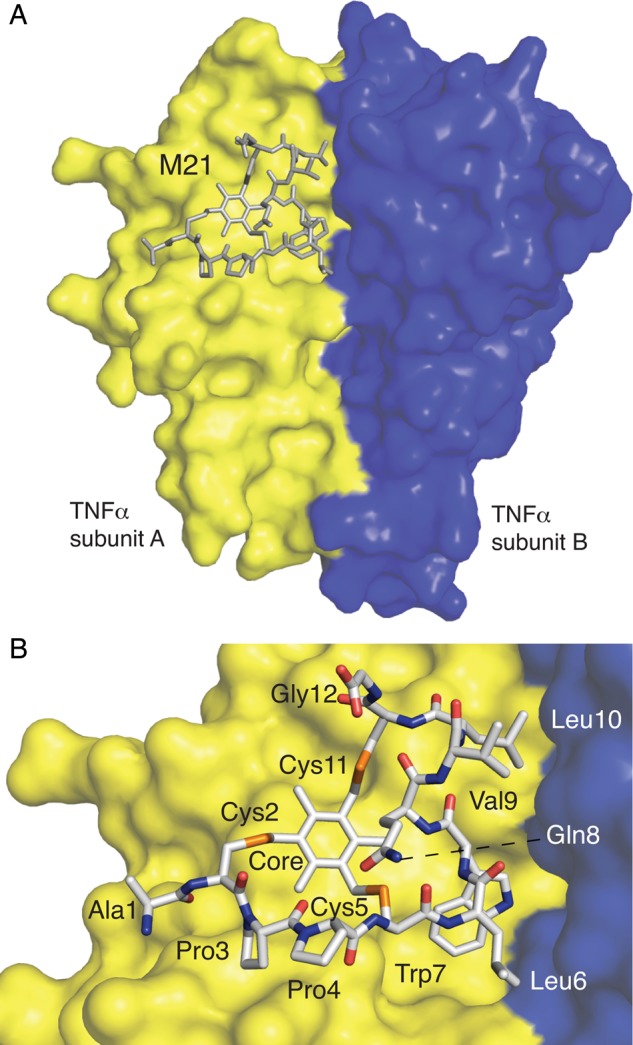
M21-TNF complex structure. (A) M21 bicyclic peptide (grey) is shown bound at the interface between 2 TNFα subunits A, B (yellow, blue) (B) as a stick diagram with elemental colours C (grey), N (blue), S (orange) and O (red).

The asymmetric unit of the crystal consists of 2 copies of a TNFα—M21 bicycle complex at a ratio of 2 : 1. The structure shows the M21 peptide bound to a dimer of TNFα subunits (A and B) (Fig. 5A and B) interacting with residues from both A and B and with an average interface area of TNFα and M21 of 231 Å^2^ (compared to 398 Å^2^ for the TNFα subunit interface).

**Fig. 6.**
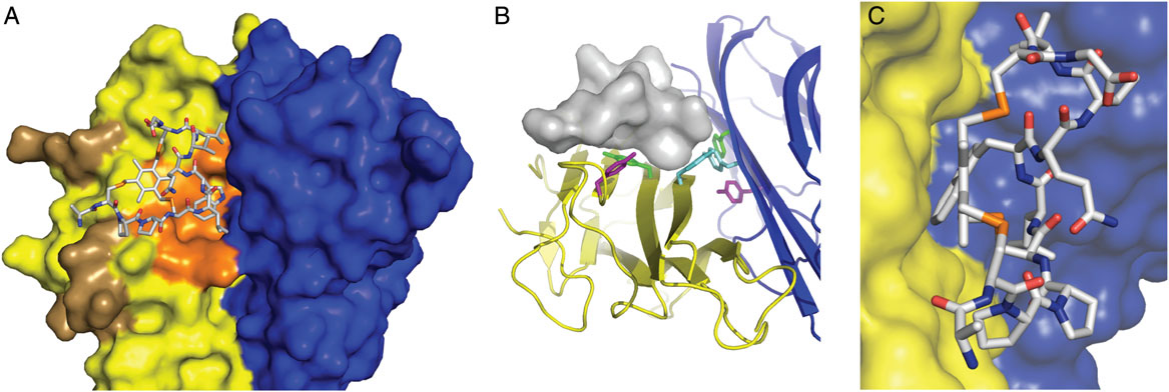
Structural aspects of M21-TNF interaction (A) M21 peptide bound to TNFα dimer (subunits A (yellow), B (blue)). Binding sites for TNF receptor R1 (brown) and SPD304 (He *et al.*, 2005) (orange) are coloured. (B) M21 peptide (shown in surface presentation) bound to TNFα dimer (ribbon diagram) illustrating interaction of M21 with Y151 (magenta), Y59 (green) and Y119 (cyan) of TNFα subunit A (yellow). (C) M21 peptide bound to TNFα dimer, shown in side-view to illustrate shape complementarity and packing of the flat organic core against a complementary region on the TNF surface.

M21 is bound at a hydrophobic epitope adjacent but non-overlapping with the TNF (R1) receptor binding site but overlapping with the SPD304 binding site (Fig. 6A). The flat surface of the M21 bicycle formed by the organic core and W7 packs against the core of the A subunit involving H15, Y59 and Y151 (Fig. 6B). The side-chains of the M21 loop 2 (residues L6, V9 and L10) make contacts with the B subunit. The only hydrogen bond observed between bicycle and TNFα (in both copies of the complex) is formed between amine group of W7 in loop2 and the hydroxyl group of Y119 side-chain in TNFα subunit B.

No other intermolecular hydrogen bonds or salt bridges are observed, suggesting that the high-affinity interaction is largely driven by hydrophobic/van der Waals interactions and shape complementarity (Fig. 6C). Thus the interaction of M21 with TNFα in many ways more closely resembles the interaction of small molecule ligands with their targets than antibody-antigen interactions.

The disassembly of TNFα trimers by M21 might occur either by induced TNFα trimer dissociation or by stabilization of a transient dimeric form. The latter is more consistent with the slow time-dependent effect of M21 action and with the inability of M21h to bind the stably trimeric transmembrane form of TNFα (tmTNF) (Supplementary Fig. S7). Moreover, the synergistic effect of SPD304 on TNF inhibition by M21h (Fig. 4) might be rationalized by SPD304's ability to destabilize TNFα trimers to the dimeric form, which are bound and stabilized by complexation with M21.

## Conclusion

In conclusion, we have developed an improved display system for the rapid generation of semi-synthetic peptide macrocycles to a range of targets. Said system has allowed the discovery of M21, a high-affinity binder to TNFα capable of inducing dissociation of the trimeric state of the pro-inflammatory cytokine TNFα, a relevant clinical target, to an inactive dimeric form. Macrocycles like M21 may be particularly suited to access cryptic epitopes only poorly druggable with antibodies and to disrupt oligomeric protein targets into inactive monomers as described herein.

## Supplementary data

Supplementary data are available at *PEDS* online.

## Funding

Funding to pay the Open Access publication charges for this article was provided by the Medical Research Council (MRC) programme grant U105178804.

## Notes

GW is a director of Bicycle Therapeutics, a company dedicated to the development of bicyclic peptides for therapy.

## Supplementary Material

Supplementary Data
