# Supplementary material for: Subunit disassembly and inhibition of TNFα by a semi-synthetic bicyclic peptide
Source: Protein Eng Des Sel. 2015 Jan 20;28(2):45–52. doi: 10.1093/protein/gzu055 (PMC4378371; doi:10.1093/protein/gzu055)
Supplement: Supplementary Data [file supp_28_2_45__index.html]

Supplementary Data 

# Subunit disassembly and inhibition of TNFα by a semi-synthetic bicyclic peptide

## Supplementary Data

Supplementary Data

**Files in this Data Supplement:**

- Supplementary Data - Docx file
