# Supplementary material for: Subunit disassembly and inhibition of TNFα by a semi-synthetic bicyclic peptide
Source: Protein Eng Des Sel. 2015 Jan 20;28(2):45–52. doi: 10.1093/protein/gzu055 (PMC4378371; doi:10.1093/protein/gzu055)
Supplement: Supplementary Data [file supp_gzu055_gzu055supp.docx]

**Supporting information:**

**Title:** Subunit Disassembly and Inhibition of TNFα by a Semi-Synthetic Bicyclic Peptide

**Authors:** Stefan Luzi, Yasushi Kondo, Elise Bernard, Lukas K. J. Stadler, Marina Vaysburd, Greg Winter and Philipp Holliger*

**Address:** MRC Laboratory of Molecular Biology, Francis Crick Avenue, Cambridge

CB2 0QH, UK

* Corresponcence should be addressed to: PH: [ph1@mrc-lmb.cam.ac.uk](mailto:ph1@mrc-lmb.cam.ac.uk)

***Figure S1 A)*** *Different TBMB organic core crosslinking protocols evaluated for phagemid particles harboring the PEP48 MDM2 binding bicyclic peptide insert. Both infectious phagemid titer (as colony forming units (cfu)) (bars) and MDM2 binding (as judged by phage ELISA signals) (dots) are compared. Protocol 1 refers to the original Heinis & Winter protocol(**Heinis et al., 2009**), while protocol 4 is the most optimized protocol for phagemid display and used herein.* ***B****) Comparison of CFU numbers (bars) and ELISA signals (dots) for PEP48 MDM2 binding bicyclic peptide crosslinked to TBMB using Protocol 4 (see A) and engineered either into the original phages with a disulfide-free pIII(**Kather et al., 2005**) (PEP48-fdg3p0ss21) or wild type pIII phages (PEP48-fdwtg3p) and compared to unmodified phages. An unmodified wild-type pIII phage with an irrelevant non-MDM2 binding insert (fdSNZIF268) is shown for comparison.*

***Figure S2: iPCR for DNA library construction***

***i)*** *The DNA library is encoded in one or both of the two primers, which can anneal to dam methylated template DNA (brown). At the end of each loop sequence, a unique “Type IIS” restriction site is encoded (e.g. BsaI, yellow) followed by a GC-rich linker segment (green).* ***ii)*** *DNA is amplified by iPCR reaction resulting in linear unmethylated dsDNA strands (black) as opposed to the methylated template strand (brown).* ***iii)*** *Restriction endonuclease DpnI specifically digests the methylated template DNA (dashed brown line), while leaving the iPCR product containing the randomized sequences intact. “Type IIS“ restriction endonuclease (e.g. BsaI) cuts both ends of the linear dsDNA product and results in two compatible sticky overhangs (yellow cap) encoding the central Cys residue of the bicycle.* ***iv-v)*** *Ligation of the digested linear iPCR product results in circular dsDNA remaining overhang encoding the central Cys residue of the bicycle. (yellow). This circular plamid is substrate for rolling circle amplification (RCA)* ***vi)*** *by phi29 DNA polymerase, resulting a hyperbranched network of DNA concatamers.* ***vii)****, which are resolved by digestion with an unique plasmid cutter and re-ligation to yield 10^2^-10^3^ fold amplified plasmid DNA, greatly boosting library size upon* ***viii)*** *transformation(**Christ et al., 2006**).*

***Figure S3: Antigen Specificity of anti-MDMX peptides***

*Binding activity of peptides selected against MDMX on either MDM2 (orange bars), MDMX (light blue bars), TNFα (red bars) or Streptavidin (green bars) is compared by phage ELISA. Phagemid particle titers (cfu / ml) before trypsinization are also shown (black squares). Assayed peptides comprise MDMXT1: CPASHPVCQ*FFC (Q*, amber codon, in supE strain presumed to be Q), MDMXM1: CSLSAPACFIMQ*FSC, MDMXM2: CSLSQ*LQCFFQGFTC, MDMXM3: CGCTEFECLWQGLFC and MDMXM4: CPHTALQCFSLSFHC. MDMXT1 (crosslinked to a TBMB core) shows binding to both MDMX and MDM2. In contrast, MDMXM1, 2 & 4 were specific for MDMX and showed binding only crosslinked to a TBMB-methyl core). MDMXM3 shows binding to both MDMX and MDM2 both crosslinked to TBMB-methyl as well as in an unmodified state. MDMXM3 contains four cysteines and was not cleavable by trypsin, suggesting it is folded up even in the absence of organic core crosslinking, suggesting the formation of two disulfide bridges. None of the clones showed any binding to TNFα or Streptavidin controls.*

***Figure S4: ELISA specificity tests.***

***A)*** *Bicycle-MBP fusion constructs assayed for binding to MDM2 (blue), TNFα (red) and Streptavidin (green).* ***B)*** *Binding of TNF-M9-MBP fusion to different members of the TNF superfamily. C) Competition of TNFα binding of MBP-fusion (peptides T2, M8, M9) by bicyclic peptide M9 prepared by solid-phase synthesis.*

***Figure S5: Core specificity****. (Top panel) Structures of different cores used; TBMB-methyl: 2,4,6-Tris(bromomethyl)mesitylene; TBMB: 1,3,5-Tris(bromomethyl)benzene; TBMB-ethyl: 1,3,5-Tris(bromomethyl)-2,4,6-triethylbenzene; TBAB: N,N’,N’’-(benzene-1,3,5-triyl)tris(2-bromoacetamide; TATA: 1,3,5-triacryloyl-1,3,5-triazinane. (Bottom panel) TNF phage ELISA of M9 peptide (CPPCVWQVFC) phagemid modified with different cores TBMB-methyl, TBMB, TBMB-ethyl, TBAB, TATA, not modified, or reduced (TCEP) respectively oxidized in the absence of organic cores. Only TBMB-methyl core supports TNF binding.*

***Figure S6: Cell survival experiments with the L929 bioassay****.* ***A*** *Titration of bicyclic peptides to 250pg/ml TNFα with 2h preincubation at 37˚C, 5% CO_2_.* ***B*** *Titration of bicyclic peptides to 250pg/ml TNFα with 24h preincubation at 37˚C, 5% CO_2_. Bicyclic peptide concentrations are indicated on the x-axis. Rescued cell viability is indicated on the y-axis. Cell death was measured by monitoring residual cell metabolism using a colorimetric assay (tetrazolium salt XTT). Peptides are M21h and closely related variants M22: CPWCPWQVLC, and M28: CPPCPWQALC as well as control peptides R3T1: ACIRSLSCYILGCG, PK15: CSDRFRNCPADEALC(**Heinis, Rutherford, Freund and Winter, 2009**).* ***C, D:*** *Different combinations of the small molecule SPD304(**He et al., 2005**) and M21h bicyclic peptide were applied to L929 mouse fibroblast cells with 2h (****C****) or 24h (****D****) preincubation. Rescued cell viabilities are indicated on the y-axis. Combined effects of M21h peptide and SPD304 small molecule between 12% and 19% higher than expected for simple additive effects in this concentration range indicating synergistic action on TNF*α *structure and function.*

***Supplementary Figure S7: A)*** *Electron density of M21 peptide bound to TNFα from two angles (top panel) and* ***B****) as stereo picture (bottom panels).* ***C****) Model of M21 peptide built into electron density and shown bound to TNFα with surface presentation of TNFα subunits 1 (yellow) and 2 (blue).*

***Supplementary Figure S8: A)*** *M21 peptide bound to TNFα dimer (subunits A (yellow), B (blue). Binding sites for TNF receptor 1 (brown) and SPD304(**He, Smith, Oslob, Flanagan, Braisted, Whitty, Cancilla, Wang, Lugovskoy, Yoburn, Fung, Farrington, Eldredge, Day, Cruz, Cachero, Miller, Friedman, Choong and Cunningham, 2005**) (orange) are coloured.* ***B)*** *M21 peptide (shown in surface presentation) bound to TNFα dimer (ribbon diagram) illustrating interaction of M21 with Y151 (magenta), Y59 (green) and Y119 (cyan) of TNFα subunit A (yellow).* ***C)*** *M21 peptide bound to TNFα dimer, shown in side-view to illustrate shape complementarity and packing of the flat organic core against a complementary region on the TNF surface.* ***D)*** *M21 peptide (blue) bound to TNFα dimer with hydrophobicity plotted on surface of TNFα dimer.*

***Supplementary Figure S9:*** *Fluorescent staining of tmTNF*α*-expressing Jurkat cells.* *Enbrel specifically binds to tmTNF*α*-expressing cells, as confirmed by cytometric analysis (****A****), and confocal microscopy (****D,G****). Neither the anti-TNF*α *bicyclic peptide M21h (****B,E,H****), nor the scrambled negative control bicyclic peptide R3T1: ACIRSLSCYILGCG (****C,F,I****) bind to tmTNF*α *exogenously expressed on Jurkat cells.*

**Supplementary Tables:**

**Table S1: Library compositions and sizes**

| Individual Libraries | Lib1 | Lib2 | Lib 3 | Lib 4 | LibPool1 | LibPool2 |
| --- | --- | --- | --- | --- | --- | --- |
| Loop sizes | 6+6 | 3+6 | 6+3 | 3+3 | 6+5, 6+4, 6+2, 5+5, 5+4, 5+3, 5+2, 4+6, 4+4, 4+3,  4+2 | 5+6, 2+6, 4+5, 3+5, 2+5, 3+4, 3+2, 2+4, 2+3, 2+2 |
| Library Sizes [CFU] | 2.7 x 10^9^ | 1.3 x 10^9^ | 1.7 x 10^9^ | 3.6 x 10^9^ | 1.1 x 10^10^ | 1.5 x 10^10^ |

**Table S2: X-ray Data collection (**Highest resolution shell is shown in parenthesis)

| Space group | P 3_2_ 2 1 |
| --- | --- |
| *Cell Dimensions* |  |
| a, b, c (Å) | 78.4, 78.4, 230.4 |
| α, β, γ (˚) | 90.0, 90.0, 120.0 |
| *Data Collection* |  |
| Wavelength (Å) | 0.98 |
| Resolution (Å) | 65.16 - 2.95 (3.13-2.95) |
| R_merge_^a^ | 0.109 (0.560) |
| I/sI | 7.9 (2.2) |
| Completeness (%) | 98.1 (98.3) |
| Multiplicity | 4.5 (4.4) |
| *Refinement* |  |
| Number of unique reflections | 55,721 |
| R_work_/R_free_(%) | 22.9 / 28.6 |
| Average B factors (Å^2^) | 81.9 |
| Number of atoms (nonhydrogen) | 4,629 |
| *Stereochemistry* |  |
| Rmsd bond lengths (Å)^b^ | 0.011 |
| Rmsd bond angles (˚)^b^ | 1.69 |
| Ramachandran outliers (%)^c^ | 0 |
| Ramachandran favored (%)^c^ | 98.4 |

**References**

Christ D., Famm K. and Winter G. (2006) Tapping diversity lost in transformations--in vitro amplification of ligation reactions. *Nucleic Acids Res,* **34**, e108. First published on 2006/09/02, doi: gkl605 [pii]

10.1093/nar/gkl605.

He M.M., Smith A.S., Oslob J.D., Flanagan W.M., Braisted A.C., Whitty A., Cancilla M.T., Wang J., Lugovskoy A.A., Yoburn J.C. *et al.* (2005) Small-molecule inhibition of TNF-alpha. *Science,* **310**, 1022-1025. First published on 2005/11/15, doi: 310/5750/1022 [pii]

10.1126/science.1116304.

Heinis C., Rutherford T., Freund S. and Winter G. (2009) Phage-encoded combinatorial chemical libraries based on bicyclic peptides. *Nat Chem Biol,* **5**, 502-507. First published on 2009/06/02, doi: nchembio.184 [pii]

10.1038/nchembio.184.

Kather I., Bippes C.A. and Schmid F.X. (2005) A stable disulfide-free gene-3-protein of phage fd generated by in vitro evolution. *J Mol Biol,* **354**, 666-678. First published on 2005/11/02, doi: S0022-2836(05)01204-0 [pii]

10.1016/j.jmb.2005.09.086.
